# A SecA-associated protease modulates the extent of surface display of staphylococcal protein A

**DOI:** 10.1128/jb.00522-24

**Published:** 2025-03-26

**Authors:** Muhammad S. Azam, Amany M. Ibrahim, Owen Leddy, So-Young Oh, Olaf Schneewind, Dominique Missiakas

**Affiliations:** 1 Howard Taylor Ricketts Laboratory, Department of Microbiology, The University of Chicago 456539, Chicago, Illinois, USA; University of Notre Dame, Notre Dame, Indiana, USA

**Keywords:** *Staphylococcus aureus*, protein secretion, PepV, staphylococcal protein A, YSIRK secretion motif, cross-wall

## Abstract

In bacteria, signal peptides direct pre-proteins to the SecYEG secretion channel and are typically cleaved by signal peptidases during translocation across the membrane. In gram-positive bacteria, such as *Staphylococcus aureus*, some signal peptides have a pre-translocation function. Staphylococcal protein A (SpA) carries such an atypical signal sequence, with a YSIRK/GXXS motif that directs its precursor into the cross-wall of dividing cells for subsequent anchoring by sortase A. Here, we report that PepV—a member of the M20 peptidase family which has been described as a manganese-dependent dipeptidase *in vitro*—may influence the surface display of precursors with a YSIRK/GXXS motif. SpA deposition into cross-walls was increased in Δ*pepV* bacteria. Yet, in the absence of *pepV*, neither the kinetics of signal sequence processing nor the final product of the sorting reaction was altered. In pull-down experiments, PepV was identified as a ligand of SecA. When purified PepV was incubated with SpA precursors, this interaction triggered self-cleavage of the enzyme, an unexpected activity exacerbated by the presence of a chelating agent. In agreement with this finding, a pulse-chase experiment revealed that the half-life of PepV is extended in bacteria lacking *spa*. Collectively, these data reveal a mutually inhibitory relationship between SpA precursors and PepV, the net result suggesting that while PepV may reduce the surface display of SpA, SpA precursors destabilize PepV possibly to overcome such inhibition.

**IMPORTANCE:**

The “signal hypothesis” proposed that N-terminal sequences of secretory proteins contain targeting cues directing nascent polypeptides to the endoplasmic reticulum. This concept was later confirmed as broadly applicable, even to prokaryotes with a single membrane. In gram-positive bacteria, signal sequences bearing the YSIRK/GXXS motif are necessary and sufficient to direct precursors to septal membranes. However, trans-acting factors involved in this spatially restricted targeting remain largely unknown. Here, we identify a member of the M20 metalloprotease family as a potential contributor to the septal surface display of proteins containing YSIRK/GXXS signal peptides.

## INTRODUCTION

The surface of *Staphylococcus aureus* displays approximately 24 proteins, although the exact count varies by strain (1). These proteins serve essential biological functions, including acquiring iron from hemoglobin, adhering to and invading host cells and tissues, and evading immune defense mechanisms to facilitate colonization and infection (2, 3). A majority of these proteins become covalently attached to the cell wall (CW) through the action of enzymes called sortases. Cell wall-anchored proteins possess both an N-terminal signal peptide for secretion and a C-terminal signal with an LPXTG-like motif, hydrophobic region, and positively charged tail (4–6). Once the signal peptide is removed, the protein remains membrane (Mb) bound via its C-terminal hydrophobic domain until sortase cleaves the protein and captures it as a thioester-linked acyl-enzyme (7, 8). This complex is resolved upon nucleophilic attack by the free amino group of glycine present in lipid II peptidoglycan precursors; the product of this reaction is then incorporated into the growing cell wall (4, 5). Staphylococci distribute proteins like staphylococcal protein A (SpA) and other sortase A-anchored products across their bacterial surface by incorporating secreted polypeptides directly into newly synthesized cross-walls (9, 10). The presence of the conserved YSIRK/GXXS motif in the signal sequence distinguishes those precursors directed to the dividing cross-walls, while surface proteins carrying the canonical signal peptide are deposited at the cell poles (9). Of note, the YSIRK/GXXS motif is found in surface proteins of other gram-positive organisms, where it plays a similar targeting function, at least in streptococci (11, 12). Despite the importance of surface proteins in staphylococcal and streptococcal diseases, our understanding of the specific factors and mechanisms governing their localized presentation and the significance of the signal peptide remains limited.

Recently, we conducted a genetic study to identify novel factors that may impact the production, processing, or targeting of proteins with a YSIRK/GXXS motif in the signal sequence. A collection of thermo-sensitive (TS) mutants was first isolated and screened for altered production or secretion of proteins at non-permissive temperatures using immunoblotting (13). One such TS isolate identified a thermosensitive *secA* allele. Another TS isolate was found to carry a substitution in a gene encoding the dipeptidase PepV (13). Unfortunately, this isolate was lost during subsequent passages and could not be studied further. The loss may have been caused by another mutation disrupting the *mutS* gene (14). Subsequently, PepV was also identified by mass spectrometry as a possible ligand of SecA in cleared preparations of *S. aureus* (13). Here, we generate a mutant lacking *pepV* and report that the display of both SpA and clumping factor A (ClfA), two proteins with YSIRK/GXXS motif, is increased in Δ*pepV* bacteria. We confirm that PepV co-purifies with SecA. Additional experiments rule out any contribution of PepV to the processing of SpA by signal peptidase or sortase A. *In vitro* experiments reveal that, with a chelator, purified PepV undergoes proteolysis in the presence of SpA precursor but not in the presence of mature SpA. Collectively, our data reveal an unconventional autocatalytic activity for a dipeptidase that may, in turn, modulate the surface display of some surface precursors endowed with the YSIRK/GXXS motif.

## RESULTS

### Bacterial *pepV* orthologs

**Fig 1:**
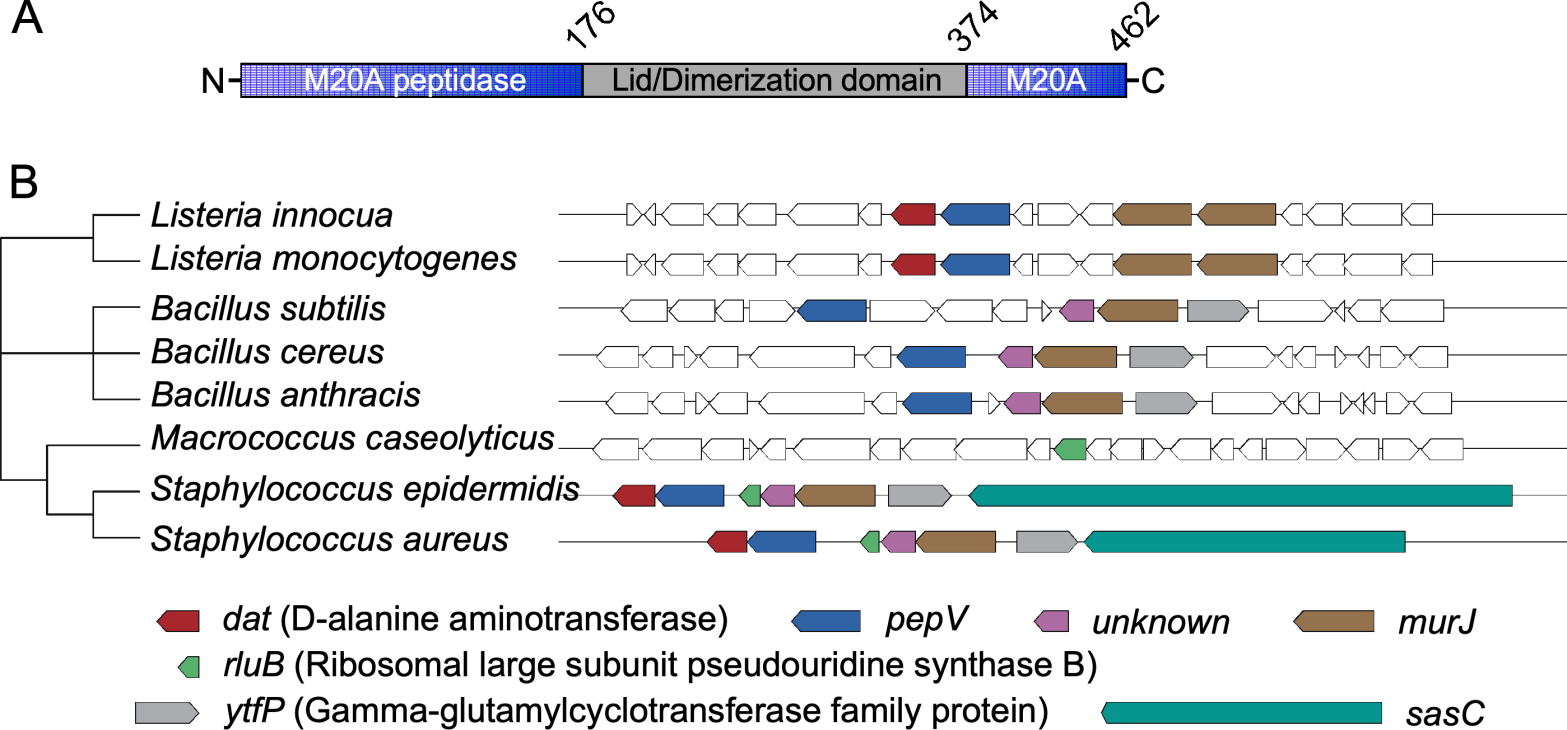
Schematic representation of PepV and the genomic neighborhood of *pepV*. (A) Linear diagram of *S. aureus* PepV with the M20A peptidase domain flanking the lid/dimerization domain. (B) Genomic loci of *pepV* according to the National Center for Biotechnology Information taxonomy database. MultiGeneBlast was used to identify multigene modules surrounding the *pepV* gene in representative species.

In *S. aureus*, PepV was identified via labeling with a small synthetic β-lactam probe developed for the identification of factors contributing to methicillin resistance (15). While its involvement in antibiotic resistance was not confirmed *in vivo*, purified PepV was shown to cleave penicillin and other dipeptide substrates *in vitro* (15). Furthermore, the protein was shown to adopt the two-domain structure of metalloproteases of the M20 family with a conventional catalytic domain and a second domain that serves as a lid but in some instances may promote dimerization defining monomeric and homodimeric enzymes, respectively (Fig. 1A) (16). Metalloproteases of the M20 family hydrolyze diverse substrates while retaining a similar active site conformation with very little sequence identity (16, 17). Indeed, a conventional local alignment search using BLASTP failed to identify PepV-like proteins in the proteome of *S. aureus*. Yet, an InterPro query using the IPR002933 (M20 protease domain) retrieved nine proteins with the following annotations or names: (i) peptidase M20 dimerization domain-containing proteins: Q2FVT7, Q2FY59, Q2G0M9, and Q2G1P6; (ii) peptidase M20 domain-containing protein 2: Q2FWC4; (iii) uncharacterized hydrolase SAOUHSC_01399: Q2FYN6; (iv) peptidase T: Q2G064; (v) probable succinyl-diaminopimelate desuccinylase: Q2FWN8; and (vi) PepV: Q2FXH9.

In *S. aureus*, the *pepV* gene is found near *sasC*, a gene encoding a cell wall-anchored protein that bears a YSIRK/GXXS signal peptide. *sasC* has been implicated in intercellular adhesion and biofilm accumulation (18) but shares no homology with genes outside of the staphylococci (Fig. 1B). *S. aureus pepV* is located downstream of the lipid II flippase *murJ* and is operonic with D-alanine aminotransferase (*dat*), encoding D-alanine aminotransferase, an enzyme involved in the synthesis of D-alanine and D-glutamate, two amino acids essential for cell wall synthesis (19, 20) (Fig. 1B). Using the Interpro and Uniprot databases, we observed proximity between *murJ*, *pepV*, and *dat* in many representative genomes but no strict association with proteins containing a YSIRK/GXXS motif that are otherwise found in the *Bacilli*, *Lactobacillales*, and *Bacillales* orders (Fig. 1B).

Using SHOOT (21) and the InterPro database (22), we found that PepV orthologs can be grouped into four clades (Fig. S1). *S. aureus* PepV clusters within clade A (red), along with orthologs from other Firmicutes (Fig. S1). Clade A is the most diverse, with some proteins containing additional domains besides the M20 catalytic and lid/dimerization domains. Conversely, the larger clade D (blue) with orthologs from *Actinobacteria* and *Bacteroidetes* phyla is the least diverse (Fig. S1). Interestingly, several clade A orthologs, including *S. aureus* PepV, contain a longer lid/dimerization domain (Fig. S1). We also noticed that a few orthologs have predicted signal peptides. This prediction has been validated for *Streptococcus gordonii* PepV, which was found in the extracellular milieu of bacterial cultures (23).

### Subcellular localization of PepV

**Fig 2:**
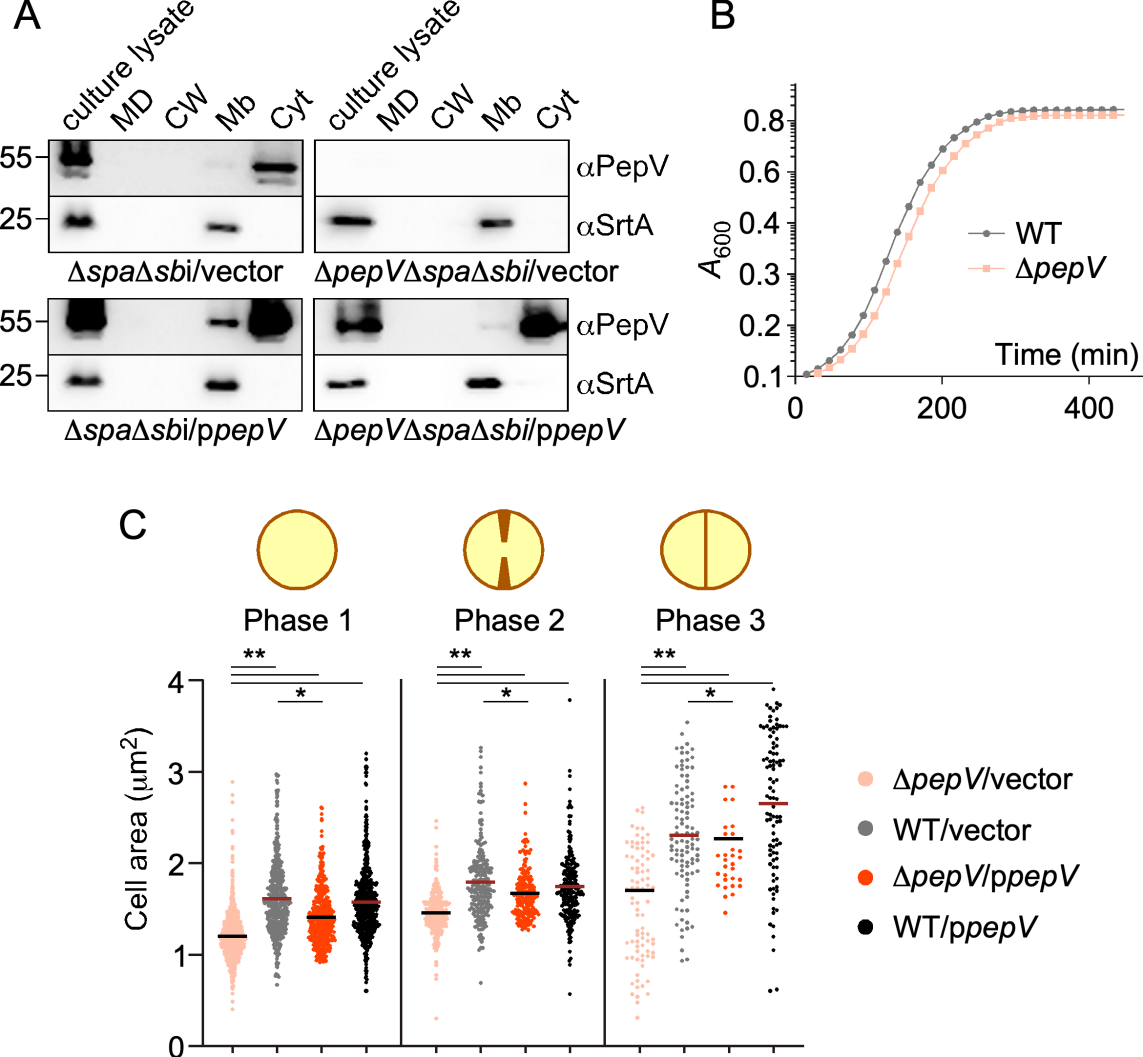
Identification of the PepV protein and growth of Δ*pepV* bacteria. (A) Bacterial cultures were lysed (culture lysate) or fractionated into medium (MD), cell wall (CW), membrane (Mb), and cytoplasm (Cyt). Proteins in all fractions were precipitated using trichloroacetic acid, separated by SDS-PAGE, and transferred to nitrocellulose membranes for immunoblotting using αPepV and αSrtA rabbit polyclonal sera. Numbers to the left of immunoblots indicate the sizes of molecular weight markers in kilodalton. (B) Growth curves of WT RN4220 and Δ*pepV* strains. At time zero, overnight cultures were diluted in TBS and incubated at 37°C with shaking. At specific time points, the absorbance at 600 nm (*A*_600_) was measured and plotted against time. A representative experiment is shown. (C) Cells of *S. aureus* WT and Δ*pepV* strains carrying control vector or the complementing plasmid p*pepV* were grown to mid-exponential phase (*A*_600_ ~0.4), washed, fixed, and stained with BODIPY-FL vancomycin to capture fluorescent micrographs. Cell areas in the micrographs were quantified for three distinct morphological phases depicted above the panel as cells with no visible septa, invaginating septa, and complete septa, respectively. The automated segmentation tool, eHooke, was used for this quantification. Statistical significance was assessed using a two-tailed *t*-test. *P ≤ 0.05, **P ≤ 0.01.

An N-terminal signal sequence could not be detected for *S. aureus* PepV using SignalP (24). To further examine the synthesis and subcellular localization of PepV, an isogenic *∆pepV* mutant and a plasmidcomplemented strain (*∆pepV*/p*pepV*) were constructed in strain RN4220, herein the wild-type (WT) strain, as well as in RN4220 *∆spa∆sbi*. The latter was performed to ensure that immune signals were not contributed by SpA or Sbi (encoded by the *spa* and *sbi* genes); SpA and Sbi bind immunoglobulin non-specifically, and SpA and PepV migrate with similar mobility on SDS-PAGE. Recombinant PepV was produced using *Escherichia coli* and used to generate a rabbit polyclonal serum (αPepV). These strains and reagents were used to analyze lysates of bacterial cultures which were further fractionated into spent medium, CW, Mb, and cytosolic compartments (Fig. 2A). Proteins in all these fractions were separated by SDS-PAGE and transferred to membranes for immune detection. Immune-reactive PepV was observed in the cytosol with a minute amount in the membrane fraction, along with the well-characterized membrane protein sortase A (SrtA) used as a control (Fig. 2A, SrtA). Immune reactivity toward PepV in both the cytosol and membrane fractions was also observed in the strain lacking *pepV* but carrying a complementing plasmid (*∆pepV∆spa∆sbi*/p*pepV*) documenting complementation (Fig. 2A).

Some members of the M20 metalloprotease family act as dipeptidases to provide amino acids for growth (17, 25), but we would not expect such limitation in rich medium in the absence of *pepV*. Nonetheless, we noted a small lag in absorbance at 600 nm (*A*_600_) in cultures of *∆pepV* bacteria as compared to RN4200 (Fig. 2B). Cells in cultures at the mid-exponential phase were stained with BODIPY-vancomycin to better visualize peptidoglycan using confocal microscopy. This analysis revealed that *∆pepV* cells appear slightly smaller than WT bacteria; size reduction was calculated by measuring the BODIPY-vancomycin staining within the cross-sectional area of single cocci and cells in the process of dividing (Fig. 2C). We speculate that this cell size reduction accounts for the lag in absorbance observed during the growth curve analysis (Fig. 2B).

### PepV is a modulator of SpA surface display

**Fig 3:**
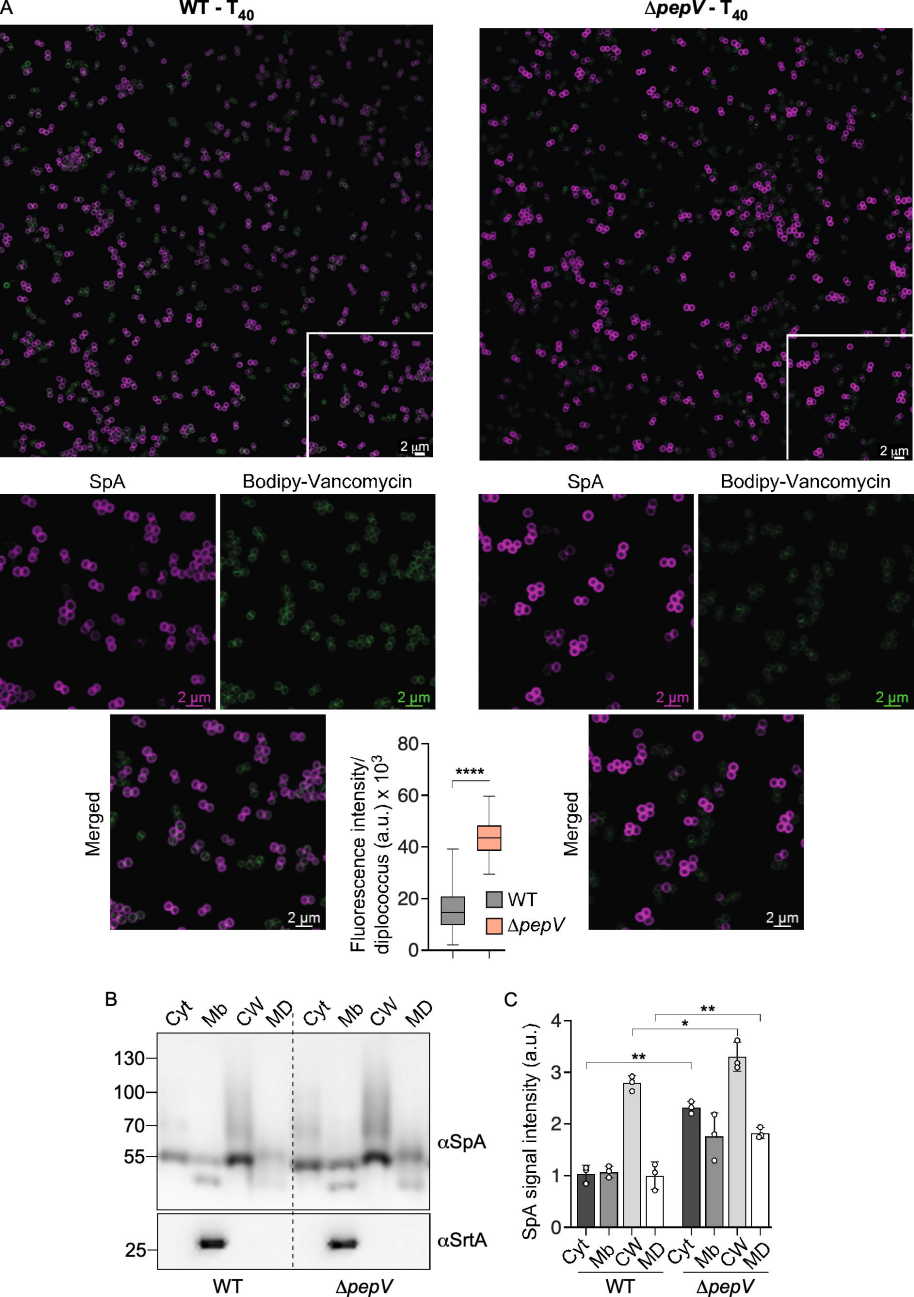
Increased SpA display in the absence of *pepV*. (A) Representative confocal micrographs of wild-type (WT) and ΔpepV bacterial cells that underwent trypsin treatment to eliminate surface-exposed proteins followed by 40 min incubation with trypsin inhibitor (T_40_) to monitor newly synthesized SpA. Samples were incubated with BODIPY-FL vancomycin (green) to stain the bacterial cell wall and with SpA-specific monoclonal antibodies followed by Alexa Fluor 594-conjugated secondary antibodies (magenta). Areas delineated with a white square are enlarged, and green, magenta, and merged channels are shown independently. Relative SpA fluorescence intensities displayed on the surface of WT and Δ*pepV* diplococci are shown as box-and-whisker plots, with the bottom, middle, and top lines of the box representing the first quartile, median, and third quartile. The whiskers extend from the minimum to the maximum data values. Statistical significance was assessed using a two-tailed *t*-test. ****P ≤ 0.0001. (B) Cultures of *S. aureus* WT and Δ*pepV* strains were fractionated into cytoplasm (Cyt), membrane (Mb), cell wall (CW), and medium (MD) for immunoblotting using αSpA, as described in Fig. 2A. Numbers to the left of immunoblots indicate the sizes of molecular weight markers in kilodalton. (C) Quantification of SpA signals as shown in panel B. The standard error of the mean was derived from three experiments, and data were analyzed with two-way analysis of variance. *P < 0.05, **P < 0.01.

In earlier studies, we used microscopy to visualize the secretion and anchoring of cell surface proteins in the envelope of *S. aureus* (9, 10). In this approach, cells are first treated with trypsin to shave all proteins from the bacterial surface followed by either 20 min incubation with trypsin inhibitor (T_20_) or 40 min incubation with trypsin inhibitor (T_40_) in the presence of a trypsin inhibitor before staining with BODIPY-vancomycin and antibodies (9, 10). Here, cells were stained with a monoclonal antibody against SpA (αSpA) and Alexa Fluor (AF)594-conjugated secondary IgG. At T_40_, a time equivalent to roughly two cell division cycles, the total distribution of SpA over the envelope was unaffected in cells lacking *pepV* (*∆pepV*); however, the fluorescence signal of SpA was significantly increased in *∆pepV* as compared to WT cells (Fig. 3A). Immunoblot analysis of fractionated cells also revealed increased amounts of SpA in the cytosol, cell wall, and medium fractions of exponentially grown cultures of *∆pepV* as compared to WT bacteria (Fig. 3B).

**Fig 4:**
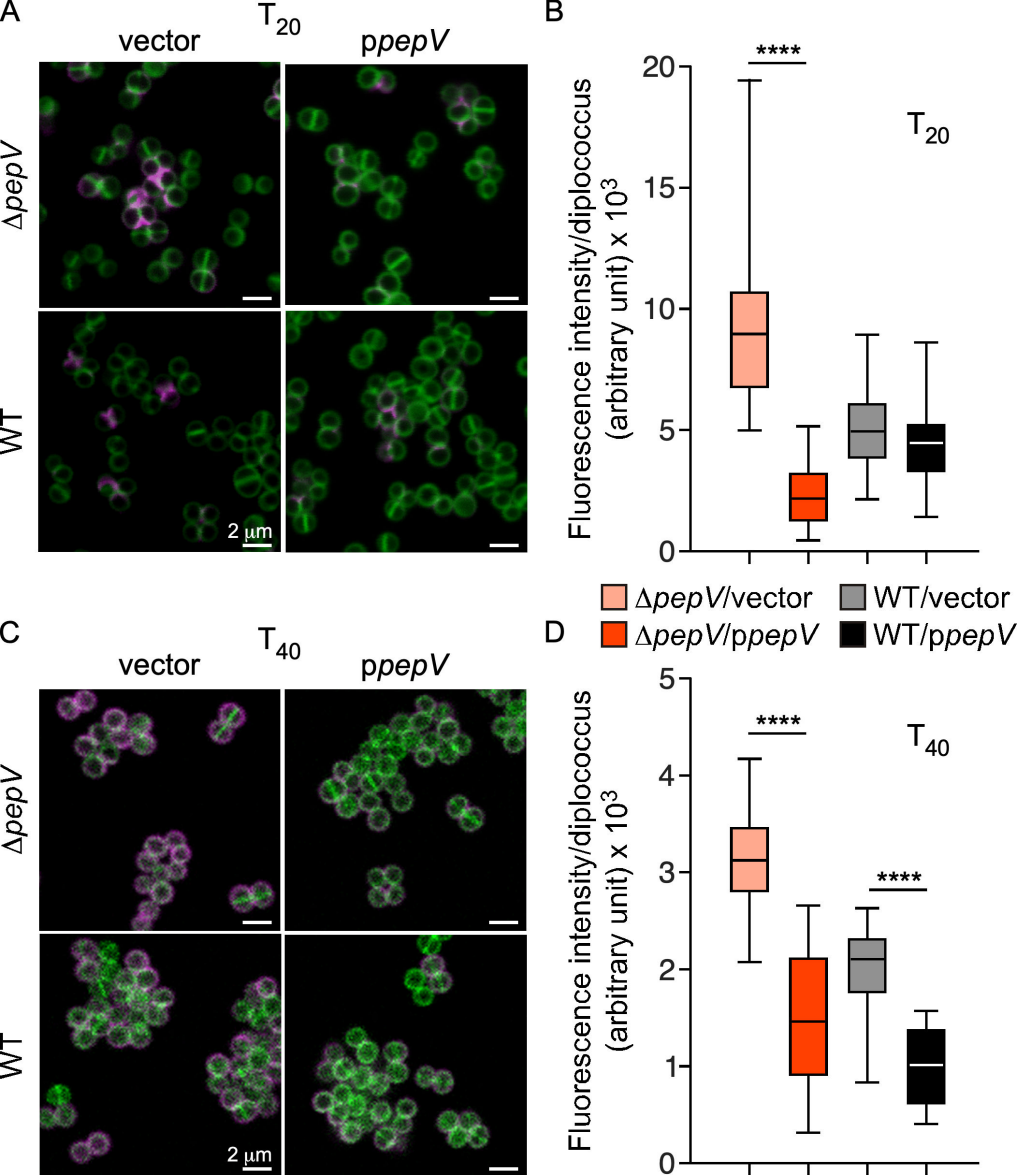
Overcomplementation by plasmid-encoded *pepV* reduces SpA display in the envelope of both WT and ΔpepV bacteria. (A and C) Representative micrographs of bacterial cells that underwent 20 min incubation with trypsin inhibitor (T_20_, A) or 40 min incubation with trypsin inhibitor (T_40_, C). Cells were then stained with BODIPY-FL vancomycin (green) and SpA-specific monoclonal antibody followed by Alexa Fluor 594-conjugated secondary antibodies (magenta). (B and D) Surface deposition of proteins in micrographs was measured for *n* = 30 diplococci from two independent experiments. Data are displayed as a box plot. Statistical analysis was performed using an unpaired *t*-test. ****P ≤ 0.0001. Bacterial strains included Δ*pepV* as well as WT bacteria carrying an empty vector (vector) or plasmid-encoded *pepV* (p*pepV*); the WT strain was included to examine the impact of *pepV* overexpression.

Next, a complementation study was performed by expressing *pepV* on a plasmid. Representative images captured at 20 and 40 min following trypsinization are presented in Fig. 4. Ectopic expression of *pepV* in the mutant (*∆pepV*/p*pepV*) resulted in reduced SpA staining at both T_20_ and T_40_ as compared to *∆pepV* or WT bacteria with vector control (Fig. 4). Of note, the T_20_ and T_40_ images were not captured on the same days; thus, fluorescence signals cannot be compared directly between the two time points. The T_20_ images further demonstrate that septal secretion was not affected in the absence of *pepV*; SpA molecules appeared along the Y or X shapes of splitting cross-walls in both WT and *∆pepV* cells at T_20_ (Fig. 4A). Interestingly, plasmid expression of *pepV* also resulted in reduced SpA staining at T_40_ in WT cells as compared to WT with vector control (Fig. 4C and D). Similarly, reduced staining was also observed for ClfA, another sortase A-anchored protein bearing a YSIRK/GXXS motif (Fig. S2) but not for SasF that lacks a YSIRK/GXXS motif (Fig. S3). For these experiments, images were captured using *∆spa∆sbi* bacteria to avoid non-specific immune signals contributed by SpA or Sbi (Fig. S2 and S3).

### PepV does not appear to impact pre-protein processing or anchor peptide composition

**Fig 5:**
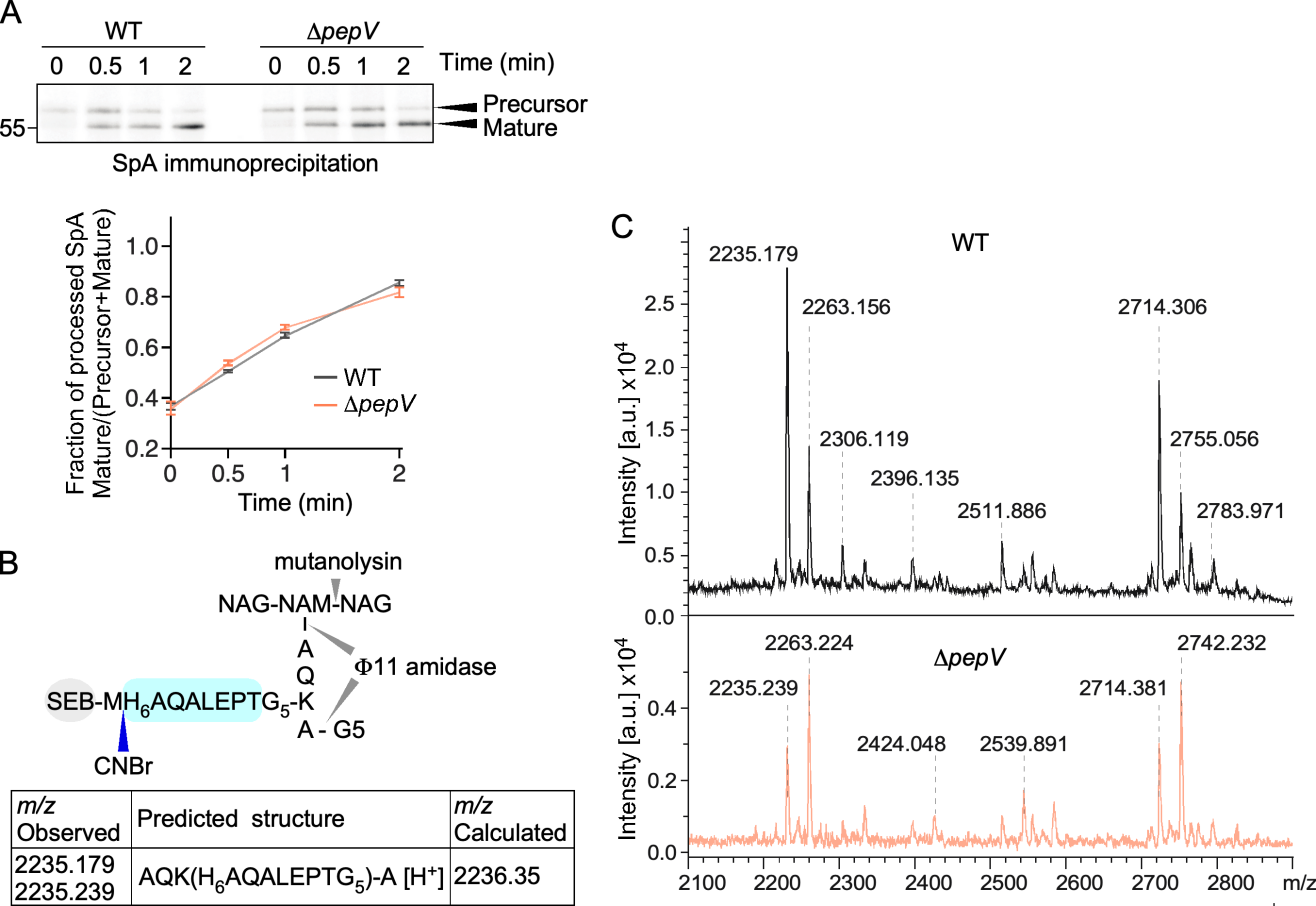
PepV deletion does not affect signal peptide processing or anchor peptide composition. (A) Processing of precursor to mature SpA was observed by pulse labeling staphylococcal cultures with [^35^S]methionine for 1 min and quenching all further incorporation by the addition of chase solution. At the indicated time intervals, aliquots of labeled culture were withdrawn, digested with lysostaphin, TCA precipitated, and subjected to immunoprecipitation with αSpA antibodies. Samples were separated on 12% SDS-PAGE, and signals were captured with PhosphorImager. The numbers to the left of the autoradiograph indicate the size of a molecular weight marker in kilodalton. The conversion of precursor to mature species was plotted as a ratio (mature/precursor + mature); the intensity of radioactive species was quantitated using ImageJ software from three independent experiments. (B) Schematic representation of the recombinant surface protein staphylococcal enterotoxin B (SEB)-single methionine followed by six histidines (MH_6_)-cell wall sorting (CWS) cross-linked to peptidoglycan. The C-terminal anchor peptides harboring cell wall structures can be purified over nickel-NTA Sepharose after treatment of peptidoglycan with mutanolysin and Φ11 hydrolase and cyanogen bromide (CNBr) cleavage at the methionine. The peptidoglycan is depicted with *N*-acetylglucosamine (NAG) and *N*-acetylmuramic (NAM) acid substituted with peptide AQKA and pentaglycine (G5) cross-links. (C) Matrix-assisted laser desorption/ionization–time of flight spectra of H6-CWS anchor peptides purified from WT (top) and ΔpepV (bottom) bacteria. Major *m*/*z* values are depicted with a diagram of the assigned structure for the observed *m*/*z* of 2,235.179 (WT) and 2,235.239 (Δ*pepV*) shown in panel B.

SpA is synthesized in the cytoplasm as a precursor bearing an N-terminal signal peptide for translocation across the plasma membrane and a C-terminal sorting signal for attachment to peptidoglycan by sortase A. Signal peptides share little sequence similarity but have characteristic features including a length range of 16–30 amino acids and a tripartite architecture comprising a positively charged *n*-region, a central hydrophobic *h*-region, and a *c*-region with the cleavage site for signal peptidases (26, 27). The SpA signal sequence is 34 residues long; the *n*-region is made of 11 residues instead of 5 and immediately followed by the YSIRK/GXXS motif. Our earlier findings suggested that this segment could undergo pre-processing in the cytosol; thus, we wondered if PepV may play any role in this process (10). Signal sequence processing is a very transient process, coupling translation and translocation tightly, but conversion from precursor to mature proteins can be captured in a pulse-chase experiment. WT and *∆pepV bacteria* were suspended in a minimal medium, and newly synthesized polypeptides were labeled by the addition of [^35^S]methionine for 1 min before the addition of an excess of non-radioactive methionine to quench all further incorporation of [^35^S]methionine. At timed intervals, all cellular processes were stopped by trichloroacetic acid (TCA) precipitation. Samples were incubated with lysostaphin and solubilized SpA molecules immunoprecipitated for visualization using a PhosphorImager after separation on SDS-PAGE (Fig. 5A). We observed no major difference in the conversion of precursor to mature SpA_ED_ in the absence of PepV nor did we detect intermediate species that would suggest additional processing by other enzymes (Fig. 5A). Of course, such species could be extremely transient and only captured in a mutant lacking the bona fide factor responsible for such processing. It should be noted that intermediate species have only been observed with a shorter, plasmid-encoded version of SpA known as SpA_ED_ (see below) (10).

Next, we asked if PepV may affect the anchored products of the sorting reaction. This line of investigation was prompted by the knowledge that sortase A has two substrates, the pre-protein and lipid II, i.e., the peptidoglycan building unit with terminal D-alanine-D-alanine residues and the fact that *pepV* is operonic with *dat* (Fig. 1). To determine the composition of anchor peptides, WT and *∆pepV* strains were transformed with plasmid pHTT4, which encodes the protein hybrid staphylococcal enterotoxin B (SEB)-single methionine followed by six histidines (MH_6_)-cell wall sorting (CWS) composed of SEB with the N-terminal signal peptide and C-terminal CWS signal of SpA (Fig. 5B). When produced in *S. aureus*, SEB-MH6-CWS is secreted and linked to peptidoglycan by sortase A (28). SEB-MH6-CWS was solubilized from peptidoglycan using mutanolysin and ϕ11 hydrolase and purified over nickel-charged (Ni-NTA) affinity resin (Fig. 5B). Since MH6 marks the fusion site between SEB and CWS (28), treatment with cyanogen bromide can be used to liberate C-terminal anchor peptides for matrix-assisted laser desorption/ionization–time of flight (MALDI-TOF) spectrometry analysis (Fig. 5C). This spectrometry analysis did not reveal any difference between WT and *∆pepV* preparations, and the presence of ions with *m*/*z* values of 2,235 and 2,263 in both samples is consistent with the calculated mass of anchor peptides harboring one pentaglycine cross-bridge and the predicted structure [NH_2_-AQK-(NH_2_-H_6_AQALPET-G_5_)-A-COOH] in protonated and formylated forms, respectively, with the latter being caused by the trifluoroacetic acid (TFA) treatment (Fig. 5B and C). These observations suggest that PepV does not alter the composition of the final product of the sortase A reaction.

### Pull-down experiments identify interactions between SecA, PepV, and SpA

**Fig 6:**
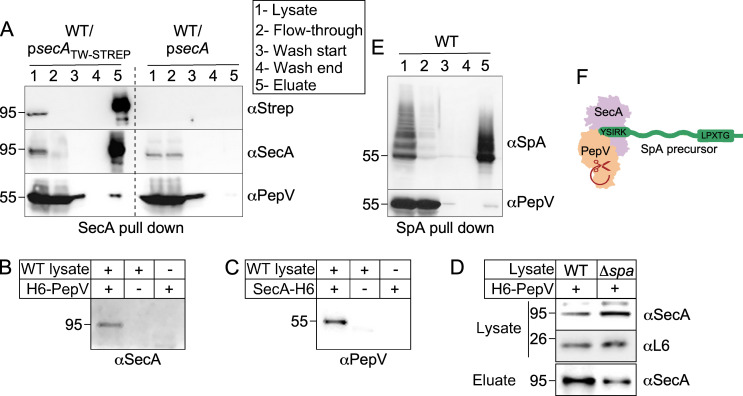
Pull-down experiments identify interactions between PepV, SecA, and SpA. (A) SecA pull-down experiment. Clarified lysates of WT carrying p*secA*_TW-STREP_ or p*secA* were passed over Strep-Tactin Sepharose resin. Aliquots from lysate, flow-through, and washes (beginning and end) were separated by SDS-PAGE prior to immunoblotting with antibodies and sera against the Strep tag (aStrep), SecA, and PepV. Eluate samples were concentrated 10 times over lysate. (B–D) Six-histidine-tagged PepV (H6-PepV) and six-histidine-tagged SecA (SecA-H6) pull-down experiments. Ni-NTA beads were preloaded with or without H6-PepV (B and D) or SecA-H6 (C). Cleared lysates of WT (B–D) and Δ*spa* (D) strains were passed over the beads. Following extensive washes, beads were boiled and eluted materials were separated on SDS-PAGE for immunoblotting with αSecA and αPepV polyclonal sera. Immunoblots of cleared lysates were also probed with αSecA and αL6 to document that the same amounts of ligands were loaded over the beads (D). (E) SpA pulldown experiment. Clarified lysates of WT bacteria were passed over IgG Sepharose. Sample aliquots were collected as in panel A and analyzed using αSpA antibodies and αPepV polyclonal serum. Numbers to the left of immunoblots indicate the sizes of molecular weight markers in kilodalton. (F) A model showing that the PepV protease associates with the SecA-SpA precursor to form a complex. Association between SpA precursor and PepV may lead to autocatalysis of PepV.

We have previously determined and validated that SecA is required for the secretion of precursors featuring the YSIRK/GXXS signal peptide (10, 13). Tandem mass spectrometry identified peptide fragments matching PepV in a pull-down experiment whereby SecA with the N-terminally fused twin-Strep-Tactin sequence (SecA_TW-STREP_) was purified over Strep-Tactin beads from lysates of *S. aureus* (13). This experiment was repeated here using WT RN4220 transformed with plasmid-borne SecA (p*secA*) or SecA_TW-STREP_ (p*secA*_TW-STREP_). Briefly, cells were harvested from bacterial cultures grown at 37°C to mid-exponential phase and treated with lysostaphin in an isotonic sucrose buffer. The resulting protoplasts were collected by centrifugation and lysed in the presence of n-dodecyl-β-D-maltoside to obtain soluble proteins from both the cytoplasmic and membrane fractions. Clarified preparations were passed over the Strep-Tactin beads, and eluted materials were subjected to immunoblot analysis. This experiment confirmed the presence of both SecA and PepV in the bacterial sample that carried p*secA*_TW-STREP_ but not p*secA*, i.e., untagged SecA (Fig. 6A).

Next, we sought to employ a reciprocal approach by tagging PepV in *S. aureus*. However, despite multiple attempts, we were unable to express epitope-tagged PepV in *S. aureus*. Instead, N-terminal six-histidine-tagged PepV (H6-PepV) or C-terminal six-histidine-tagged SecA (SecA-H6) from *E. coli* were incubated with *S. aureus* clarified preparations obtained as described above. Following purification over Ni-NTA beads, eluates were subjected to Western blot analysis (Fig. 6B and C). Control experiments indicated that untagged SecA and PepV did not interact with Ni-NTA beads non-specifically or that sera against SecA and PepV did not cross-react (Fig. 6B and C). Consistent with our observations, we detected PepV in the eluate of SecA-H6 incubated with *S. aureus* (Fig. 6B). Conversely, H6-PepV retained SecA from clarified preparations (Fig. 6C). When a clarified preparation from the *∆spa* strain was passed over H6-PepV bound to Ni-NTA, it was noted that a lower amount of SecA (~30% less) was eluted from the beads as compared to when the extract was prepared from WT RN4220 (Fig. 6D). This result indicates that the interaction between PepV and SecA may depend on SpA. Next, clarified preparations from WT RN4220 were incubated with IgG-Sepharose beads to pull down SpA. Western blotting identified both SpA and PepV in eluate fractions (Fig. 6E). Arguably, the amount of PepV pulled down with SpA is minimal but reproducible in two independent experiments and in agreement with the next experiments, suggesting that SpA precursors may destabilize PepV. Thus, together these findings suggest that PepV, SpA, and SecA may form a complex in the cell (Fig. 6F).

### Self-cleavage of PepV is triggered by metal chelation and the presence of SpA precursor

**Fig 7:**
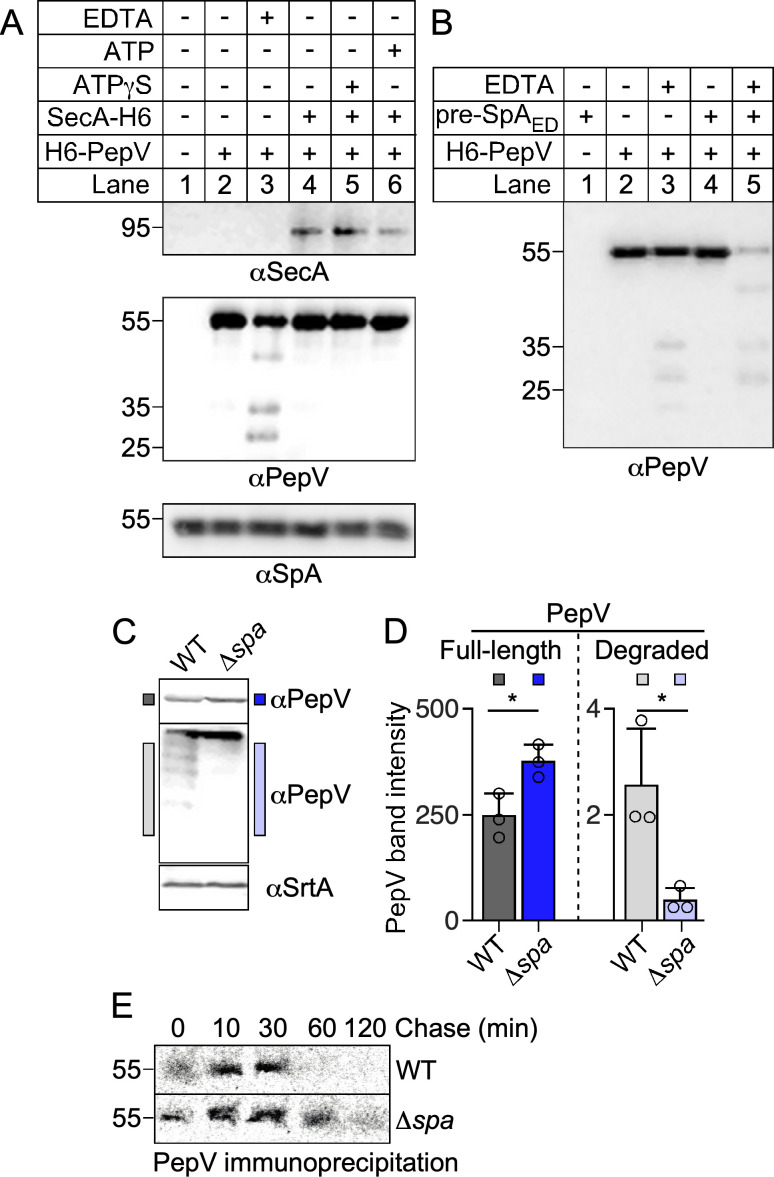
The SpA_ED_ precursor, pre-SpA_ED_, stimulates self-degradation of PepV *in vitro*. (A) H6-PepV, suspended in a buffer containing *in vitro* translated pre-SpA_ED_, was incubated for 16 h at 37°C, along with SecA-H6, EDTA, ATP, and the non-hydrolysable analog ATPγS, as indicated. Reaction products were identified by immunoblotting using polyclonal sera specific for SecA, SpA, and PepV. (B) *In vitro* translated pre-SpA_ED_ was incubated with H6-PepV and EDTA as indicated on the figure. Reaction products were identified as described in panel A. (C and D) Cultures of WT and Δ*spa* strains were lysed with lysostaphin and precipitated with TCA. Solubilized proteins in sample extracts were identified by immunoblotting using polyclonal sera specific for PepV and SrtA, the latter serving as a loading control. Two immunoblots are shown for PepV with the bottom cut right at the PepV band and overexposed to better visualize PepV degradation products. A quantification of PepV immune signals from three independent experiments is shown in panel D; statistical significance was assessed using a two-tailed t-test (*, p ≤ 0.05). (E) Pulse-chase analysis comparing the stability of PepV in WT and Δ*spa* strains. Samples were pulsed with [^35^S] methionine/cysteine for 2 min and then chased with excess non-labeled amino acids. Aliquots were removed at the indicated time intervals for immunoprecipitation with a PepV-specific polyclonal serum. An autoradiography of a representative experiment is shown. Numbers to the left of immunoblots and autoradiograph indicate the sizes of molecular weight markers in kDa.

We noted above that the YSIRK/GXXS motif could serve as a recognition site for a cytosolic protease but did not find evidence for such species in our pulse-chase experiment. To further examine whether PepV may cleave YSIRK/GXXS signal peptides, pre-SpA_ED_, a truncated SpA protein with the YSIRK/GXXS signal peptide, followed by the first two IgG-binding domains E and D, was used as a substrate *in vitro*. The pre-SpA_ED_ precursor was produced using a cell-free translation system and purified over IgG-Sepharose beads. We chose this substrate for its shorter size to ensure the fidelity of the *in vitro* translation system and because, when overexpressed in WT bacteria, an immune reactive species migrating between pre-SpA_ED_ and SpA_ED_ is observed, albeit faintly in immunoblots (10, 13). In the first experiment, pre-SpA_ED_ was incubated with purified PepV or with both purified PepV and SecA, as well as ATP and non-hydrolysable ATP. As a control, EDTA was also added to chelate the enzyme co-factor and inhibit PepV activity. We did not observe any change in the migration profile and immune reactivity of pre-SpA_ED_ and SecA. Instead, a distinct cleavage of PepV was observed in the presence of EDTA alone (Fig. 7A, lane 3). In the next experiment, PepV was incubated with either EDTA, pre-SpA_ED_, or both. As before, the addition of EDTA alone promoted self-cleavage but was exacerbated in the presence of the pre-SpA_ED_ precursor (Fig. 7B). We wondered if SpA precursors may also enhance the self-cleaving activity of PepV *in vivo*. We reasoned that if this is the case, the total amount of PepV would be higher in *∆spa* bacteria. Indeed, an immunoblot analysis revealed a slightly greater abundance of PepV when the *spa* gene was absent (Fig. 7C). When the bottom section of the blot was separated and incubated with αPepV sera (this was done to eliminate full-length PepV that preferentially adsorbs antibodies), PepV degradation products were readily observed in the WT extract (Fig. 7C and D). To further examine the stability of PepV, a pulse-chase experiment was performed by labeling WT and *∆spa* cultures with [^35^S]methionine/cysteine for 1 min. Unlabeled amino acids were added for 0, 10, 30, 60, and 120 min before sample aliquots were TCA precipitated for immunoprecipitation of PepV. ^35^S-labeled PepV was detected up to the 60 min timepoint (with a faint signal still present at 120 min) when SpA was absent. However, PepV could no longer be detected in the WT background after the 30 min chase (Fig. 7E). Thus, while surprising, together these results are consistent with the notion that the SpA precursor stimulates PepV autodegradation, leading to a faster turnover of PepV *in vivo* as modeled in Fig. 6F.

## DISCUSSION

Signal peptides discriminate exported proteins from those that remain in the cytosol by selectively binding and targeting their substrates to cognate translocases in the cytoplasmic membrane (29, 30). Bacterial Sec signal peptides also bear regulatory pre-targeting functions to slow down protein folding (31, 32) or by acting as allosteric activators of the Sec translocase (33). In *S. aureus* and gram-positive bacteria, the YSIRK/GXXS motif within extended signal peptides acts as a *cis* element for secretion of precursors across septal membranes (6, 9). Recently, we described the genetic requirement for SecA corroborating the notion that precursors with YSIRK/GXXS motif are exported post-translationally (10). SecA-bound precursors also interact with chaperones to remain translocation competent (30). Here, we present evidence suggesting that cytosolic PepV, a member of the M20 peptidase superfamily, may act as a modulator of SpA surface display. Our findings can be summarized as follows: (i) deleting *pepV* resulted in a net increase in the surface display of SpA and ClfA, both of which are endowed with a YSIRK/GXXS motif; (ii) in pull-down experiments, PepV was found to associate with both SpA and SecA; (iii) incubating PepV with the SpA precursor peptide, pre-SpA_ED_, enhanced PepV autodegradation *in vitro*; and (iv) conversely, deleting *spa* resulted in the increased stability of PepV. Together, these observations suggest that YSIRK/GXXS precursors may destabilize PepV, a process that may, in turn, modulate their interaction with SecA. SpA is one of the most abundant proteins in *S. aureus*. Whether other precursors containing the YSIRK/GXXS motif influence the PepV-SecA interaction or PepV stability remains to be determined. Together, these observations suggest the existence of a regulatory feedback loop between SpA and PepV: PepV hinders the surface display of SpA, while SpA precursors promote the autodegradation of PepV and counteract its inhibitory effect. This model does not rule out the possibility that PepV may play additional roles in the cell but points to an unusual function for an enzyme otherwise described as a dipeptidase (16). This model also does not rule out the possibility that PepV may directly or indirectly regulate the extent of SpA production at the transcriptional or translational levels.

PepV is identified as a member of the M20A peptidase superfamily within the MH clan, which consists of four families. Enzymes in the M20A subfamily are known for catalyzing reactions that release N-terminal amino acids, typically of a neutral or hydrophobic nature, from polypeptides (16, 34). However, the M20 family also encompasses enzymes that are not peptidases, such as the ArgE/DapE/CPG2 family, including aminoacylase, acetylornithine deacetylase, and succinyl-diaminopimelate desuccinylase (such as DapE), which removes succinate from succinyl-diaminopimelate (17, 35–37). Biochemical data are only available for some homologs. Although M20 proteases share only limited sequence similarity, their structures and the presence of two catalytic Mn^2+^ ions are highly conserved (16). Here, we find that *S. aureus* PepV may also have a self-cleaving activity. To our knowledge, such an activity has not been reported before for this family. As self-cleavage was first observed following overnight incubation with EDTA, we can rationalize our findings owing to the requirement of Mn^2+^ ions for catalysis (16). In the case of *Haemophilus influenzae* DapE, it was reported that stripping of both co-factors was met with partial success, suggesting that one of the catalytic ions binds the enzyme with high affinity (35). We can only surmise that EDTA stripping of *S. aureus* PepV occurs slowly, resulting in self-cleavage as the enzyme transitions to an apo state (16). Despite high local concentrations of reactants, self-cleavage is expected to proceed at a slower rate compared to most catalyzed reactions because of the gradual binding of the cleavage site to the active site. In line with this model, we speculate that an interaction with the SpA precursor bound to SecA could serve as a physiological trigger resulting in the self-cleavage of PepV (Fig. 6F). We cannot rule out that other interactions may also result in PepV self-cleavage.

In this study, we also investigated the impact of PepV on the final product of the sorting reaction. With the exception of the secreted glycerol ester hydrolase (Geh), all YSIRK/GXXS motif-bearing precursors are also substrates of sortase in *S. aureus* (12, 38). Two related PepV enzymes in *Lactobacillus delbrueckii* and *Lactobacillus lactis* were shown to cleave β-alanine-dipeptides (39) and alter the pool of intracellular amino acids, resulting in an unusual peptidoglycan composition (25), respectively. Although we have not thoroughly examined the peptidoglycan composition of bacteria lacking *pepV*, we observed that the structure of sortase A-anchored products was unaffected in the absence of PepV, suggesting that PepV does not impact the later sorting reaction occurring on the *trans* side of the plasma membrane. Nonetheless, we do not rule out the possibility that PepV or another factor recruited to YSIRK/GXXS precursors bound to SecA could help coordinate secretion and the availability of lipid II, the peptidoglycan biosynthesis precursor on the *cis* side of the septal membranes.

In conclusion, we propose that the cytoplasmic factor, PepV, acts as a modulator of surface display of precursor proteins with a YSIRK/GXXS motif. Our data indicate that the absence of PepV leads to an overall increase in the steady-state level of SpA, but the exact mechanism whereby PepV exerts this activity requires further investigations. For example, we have yet to explain the changes in cell area and doubling time observed for Δ*pepV* bacteria, and conversely, the slightly increased cell size observed upon overproduction of PepV. These changes correlate inversely with the abundance of SpA and could also contribute to some of the phenotypes reported here.

## MATERIALS AND METHODS

### Media, growth conditions, bacterial strains, and plasmids

Strains and plasmids used in this study are listed in Table S1, and primers are listed in Table S2. *S. aureus* strain RN4220 and derivatives were used for all experiments (40). *S. aureus* was cultured in tryptic soy broth or on tryptic soy agar plates and, when necessary, supplemented with erythromycin and chloramphenicol at a concentration of 10 µg/mL. Minimal medium lacking cysteine and methionine was prepared as described (5). *Escherichia coli* DH5a and BL21(DE3) were used for cloning and production of histidine-tagged proteins, respectively. *E. coli* was grown in lysogeny broth or Luria broth agar supplemented with 100 µg/mL ampicillin for plasmid selection and 0.5 mM isopropyl β-d-thiogalactopyranoside (IPTG) for induction of plasmid-encoded proteins. Deletion of the *pepV* gene was achieved by allelic replacement using plasmid pKOR1 (41). Briefly, three DNA fragments were amplified by polymerase chain reaction (PCR) using primer pairs upepVF/upepVR and dpepVF/dpepVR (generating two 1 kbp DNA fragments flanking the *pepV* coding sequence) and the primer pair bkpKOF/bkpKOR (to amplify the pKOR1 backbone). The three PCR products were then assembled utilizing RecA-independent recombination (28). *pepV* complementation studies were conducted by cloning PCR products amplified with primers FPepV(SacI)/RPepV(XhoI) into the shuttle vector pSEW016 with the in-built Shine-Dalgarno and promoter sequences of the *S. aureus hprK* gene (42, 43). For the production of N- and C-terminally six-histidine tagged His-PepV and SecA-His in *E. coli*, PCR products amplified with primer pairs pepV-F-XhoI/pepV-R-XhoI or OL6/OL3 were cloned in pET16b and pET15b, respectively. Plasmids p*secA* and p*secA*_TW-STREP_ expressed in *S. aureus* were as described in our accompanying paper.

### Cellular fractionation and immunoblotting

For subcellular fractionation of PepV, *S. aureus* cultures grown to an *A*_600_ of approximately 0.4–1.0 were subjected to centrifugation at 10,000 × *g* for 10 min. Supernatant fractions containing proteins secreted in the medium were transferred to new tubes. Cells were resuspended in tris-sucrose-magnesium buffer (TSM) buffer (50 mM Tris-HCl, pH 7.5, 0.5 M sucrose, and 10 mM MgCl_2_) and lysostaphin (10 µg/mL), then incubated for 1 h at 37°C to obtain protoplasts that were recovered by centrifugation at 15,000 × *g* for 10 min. Supernatants containing cell wall material were transferred to new tubes, while protoplasts were lysed through freeze-thaw cycles and subjected to 100,000 × *g* centrifugation for 40 min at 4°C to separate the cytosolic proteins (supernatant) from membrane proteins in pellets. Proteins in all fractions were precipitated with 10% trichloroacetic acid (final concentration), washed with acetone, dried, and solubilized in SDS sample buffer (62.5 mM Tris-HCl, pH 6.8, 2% SDS, 10% glycerol, 5% 2-mercaptoethanol, and 0.01% bromophenol blue). Proteins were resolved on SDS-PAGE and identified by staining gels with Coomassie brilliant blue or by immunoblotting after electro-transfer to polyvinylidene difluoride (PVDF) membrane. For western blotting experiments, PVDF membranes were blocked with a 10 mL blocking solution (5% milk solution and 70 µL of human IgG, as needed) for 30 min, exposed to primary antibodies for 1 h, followed by three 10 min washes with tris-buffered saline with Tween 20 (TBST) (50 mM Tris-HCl, pH 7.5, 150 mM NaCl, and 0.1% Tween 20). Membranes were incubated with a secondary antibody linked to horseradish peroxidase, followed by three washes with TBST and development with SuperSignal Chemiluminescent Substrate (Thermo Scientific). Experiments were conducted in triplicate and repeated at least once. Polyclonal sera used in this study were obtained in-house by immunizing rabbits with purified PepV, SecA, ClfA, ribosomal protein L6, or SrtA. Other antibodies, including secondary and anti-Strep monoclonal antibodies, were commercially obtained. SpA-specific monoclonal antibodies were as described (44).

### Protein labeling and immunoprecipitation experiments

Bacterial cultures were grown overnight in a chemically defined medium, diluted 1:20 into minimal medium lacking methionine and cysteine, and pulse-labeled at an *A*_600_ of 0.4. One milliliter of culture was labeled with 50 µCi of [35S]methionine/cysteine (Express S35 protein labeling mix, PerkinElmer Life Sciences) for 1 min (Fig. 5A) or 2 min (Fig. 7), and the incorporation of radioactive amino acids was quenched by the addition of 50 µL of chase solution (100 mg/mL casamino acids, 10 mg/mL methionine and cysteine). Cultures were incubated, and at defined intervals, 250 µL aliquots were withdrawn and precipitated with TCA. Precipitates were digested with lysostaphin (1 mL of 0.5 m Tris-HCl, pH 8.0, 100 µg/mL enzyme) for 1 h at 37°C. Digests were precipitated by adding 75 µL of 100% trichloroacetic acid, centrifuged, washed in acetone, and dried. The precipitate was solubilized in 50 µL of 0.5 M Tris-HCl, 4% SDS, pH 8.0, by heating to 95°C for 5 min. The samples were centrifuged at 13,000 × *g* for 10 min to remove any insoluble material, and 40 µL was transferred to 1 mL of radioimmunoprecipitation assay (RIPA) buffer (50 mm Tris-HCl, pH 8.0, 150 mm NaCl, 1% Triton X-100, 0.5% deoxycholate, and 0.1% SDS) containing rabbit α-PepV immune serum. Antigen-antibody complexes were captured on pre-swollen protein A CL 4B-Sepharose, washed five times in RIPA buffer, boiled in 50 µL of SDS sample buffer, and separated by SDS-PAGE. Radioactive signals were captured by scanning dried gels with an Amersham GE Typhoon PhosphorImager. All immunoprecipitation experiments were performed at least twice.

### Fluorescence microscopy

For microscopy experiments, cells from bacterial cultures were fixed and immunostained mostly as described (10, 45). Briefly, for SpA staining, cells were washed with phosphate-buffered saline (PBS), treated with 0.5 mg/mL trypsin for an hour at 37°C, and washed twice with PBS before resuspension in 900 µL of fresh tryptic soy broth (TSB) with soybean trypsin inhibitor (1 mg/mL) for 40 min at 37°C. Bacterial samples were incubated for 20 min in the presence of 2.5% paraformaldehyde and 0.006% glutaraldehyde and washed twice with PBS before they were applied to a poly-L-lysine coated glass slide. The immobilized cells were blocked with a 3% wt/vol bovine serum albumin solution in PBS and incubated for 30 min at room temperature followed by incubation with SpA-specific monoclonal antibody (44) or ClfA-specific or SasF-specific rabbit serum (laboratory collection) at 4°C overnight. Samples underwent eight washes with PBS before incubation with secondary antibody (AF94-conjugated anti-human or anti-rabbit IgG) for 3 h at room temperature. An on-slide wash was performed with PBS and incubation with BODIPY-FL vancomycin (Invitrogen) for 10 min. ProLong Diamond Antifade (Invitrogen) was used to mitigate photobleaching, and images of slides were observed and captured using a Leica Stellaris 8 Confocal microscope. All microscopy experiments were performed at least twice.

### Measuring cell size using fluorescent microscopy images

To measure the cell size of bacteria, eHooke software was used for segmentation, cross-sectional area measurements, and cell-phase estimation of vancomycin-stained cells in the mid-exponential phase (46). The segmentation results were visually inspected, and cells that were not properly segmented were excluded from the analysis. To estimate SpA intensity, measurements were manually performed using ImageJ software, with SpA intensity calculated per diplococcus (47). Diplococci were defined as two daughter cells with an invaginating or complete septum, as illustrated by phase 2 and 3 cells in Fig. 2C. For each experiment, at least two random images were acquired per sample. Statistical significance was assessed using a two-tailed *t*-test.

### Purification of proteins produced in *E. coli*

Recombinant proteins, His-PepV and SecA-His, were produced using *E. coli* BL21(DE3). Following IPTG induction of cultures, cells were sedimented (10,000 × *g* for 10 min), washed, and suspended in 15 mL of buffer A (50 mm Tris-HCl, pH 7.5, 150 mm NaCl, and 10 mM imidazole) and lysed by two passages in a French press at 14,000 lb/in^2^. Unbroken cells were removed by centrifugation (5,000 × *g* for 15 min), and crude lysates were subjected to ultracentrifugation (100,000 × *g* for 1 h at 4°C). Soluble recombinant proteins were subjected via gravity flow to chromatography on Ni-NTA resin (Qiagen) with a packed volume of 1 mL pre-equilibrated with buffer A. Columns were washed with 20-bed volumes of buffer A and eluted in 2 mL fractions with a step gradient of imidazole (20–500 mm). Aliquots of samples loaded on the column, flow-through, wash, and eluted fractions were mixed with an equal volume of sample buffer and separated on 12% or 15% SDS-PAGE. Proteins in gels were visualized by staining with Coomassie brilliant blue or by Western blotting. Fractions containing the protein of interest were dialyzed in PBS and stored at −80°C until use.

### Co-purification and pull-down experiments

For purifications of proteins produced in *S. aureus*, bacterial cells from 2 L cultures were collected by centrifugation and suspended in TSM buffer with cOmplete, Mini, EDTA-free Protease Inhibitor Cocktail (Roche) and treated with lysostaphin (10 µg/mL) for 1 h at 37°C. Protoplasts were sedimented (15,000 × *g* for 10 min at 4°C), suspended in buffer B (20 mM Tris-HCl, pH 8.0, 300 mM NaCl, 10% glycerol, and 1% n-dodecyl-β-D-maltoside) or buffer C (50 mM Tris-HCl, pH 7.6, 150 mM NaCl, 5% glycerol, and 0.05% Tween 20), and lysed by freeze-thaw cycles in liquid nitrogen. Insoluble materials were removed following centrifugation at 100,000 × *g* for 1 h at 4°C, and cleared supernatants were collected to fresh tubes. For experiments involving SecA/SecA_TW-STREP_ or SpA, cleared supernatants of cognate *S. aureus* cultures were passed over Strep-Tactin Sepharose beads or Cytiva IgG Sepharose preequilibrated with buffer B or C, respectively. Strep-Tactin Sepharose beads were thoroughly washed with buffer B, and bound proteins were eluted with 5 mM desthiobiotin. The Cytiva IgG Sepharose columns were washed with buffer C, and bound SpA was eluted by adding the SDS-PAGE sample buffer and heating at 95°C. For pull-down experiments using recombinant proteins, the cleared supernatants of *S. aureus* RN4220 WT or an isogenic Δ*spa* mutant were first incubated with purified His-SecA and His-PepV for 1 h at 4°C before loading over the Ni-NTA resin for affinity purification as described in the previous paragraph. Proteins in all samples were analyzed by SDS-PAGE or Western blotting. In some instances, samples were concentrated 10 times by precipitation with trichloroacetic acid. All co-purification and pulldown experiments were performed at least twice.

### Purification of affinity-tagged anchor peptides and analysis by matrix-assisted laser desorption/ionization mass spectrometry

WT RN4220 and ΔpepV were transformed with plasmid pHTT4 producing the protein hybrid SEB-MH_6_-CWS (48). Overnight cultures (40 mL) of bacteria were used to inoculate 2 L TSB supplemented with 10 µg/mL chloramphenicol. Cultures were grown with shaking for 5 h. Cells were collected by centrifugation, washed, suspended in 100 mL of water, extracted with 100 mL of ethanol-acetone (1:1), and incubated for 30 min on ice. The cells were collected by centrifugation, washed with 300 mL of ice-cold water, and suspended in 30 mL of 0.1 m Tris-HCl, pH 7.5, for incubation with mutanolysin (333 units/mL) for 16 h, followed by incubation with ϕ11 hydrolase (250 µg) for 16 h with rotation at 37°C. Digested samples were centrifuged at 40,000 *× g* for 30 min, and the supernatant was subjected to Ni-NTA affinity chromatography as described for recombinant proteins above. Purified Seb-MH_6_-CWS was incubated in the dark overnight with a crystal of cyanogen bromide and repurified by Ni-NTA affinity chromatography. The eluate was desalted using a C-18 matrix cartridge (Waters) and dried under vacuum, as described (48). Dried peptides were resuspended in 20 µL 50% CH_3_CN 0.1% TFA. One microliter sample was co-spotted with 1 µL α-cyano-4-hydroxycinnamic acid (10 mg/mL in 50% CH_3_CN 0.1% TFA) and allowed to dry before analysis on a MALDI-TOF instrument (Bruker) in linear positive mode (48).

## SUPPLEMENTAL MATERIAL

Supplemental tables and figures jb.00522-24-s0001.pdf Tables S1 and S2, Figures S1 to S3.

ASM does not own the copyrights to Supplemental Material that may be linked to, or accessed through, an article. The authors have granted ASM a non-exclusive, world-wide license to publish the Supplemental Material files. Please contact the corresponding author directly for reuse.
