# Supplementary material for: A SecA-associated protease modulates the extent of surface display of staphylococcal protein A
Source: J Bacteriol. 2025 Mar 26;207(4):e00522-24. doi: 10.1128/jb.00522-24 (PMC12004944; doi:10.1128/jb.00522-24)
Supplement: Supplemental tables and figures — Tables S1 and S2, Figures S1 to S3. [file jb.00522-24-s0001.pdf]

SUPPLEMENTAL MATERIAL

A SecA-associated protease modulates the surface display of Staphylococcal protein A

Muhammad S. Azam, Amany M. Ibrahim, Owen Leddy, [1] So-Young Oh, [2] Olaf Schneewind,† Dominique Missiakas#

The University of Chicago, Department of Microbiology, Howard Taylor Ricketts Laboratory, Lemont, Illinois, USA

Current addresses: [1]Department of Biological Engineering, Massachusetts Institute of Technology, Cambridge, Massachusetts, USA; [2] Cepheid, Sunnyvale, California, USA

This file contains:

- Supplementary Tables 1-2
- Supplementary Figures 1-3
- A list of references

Supplementary Table 1: Oligonucleotides used in this study.

**Oligo**	**Description**	**Sequence 5´-3´**
upepVF	Forward primer to amplify the upstream fragment	CTCGGAACCGGTACCATGACAATAAGACTGACGAAAGTATTGAAAAAGGTAAATTT
upepVR	Reverse primer to amplify the upstream fragment	TATTATTCCTCCACGTCGAAATCGACTTCCTTTTTTCTATAAGTTAAATTCTATTTTACATGA
dpepVF	Forward primer to amplify the downstream fragment	CGTGGAGGAATAATATATGGAAAAAATTTTTTTAAATGGTGAG
dpepVR	Reverse primer to amplify the downstream fragment	TTTCCCGACTGGAAACAACTGAAAATGAGAAACAAAATGGCACTTT
bkpKOF	Forward primer to amplify the pKOR1 backbone	TTTCCAGTCGGGAAACCTGTCG
bkpKOR	Reverse primer to amplify the pKOR1 backbone	GGTACCGGTTCCGAGGCTCAA
T7SpAF	Forward primer for SpAED precursor, for *in vitro* translation	TAATACGACTCACTATAGGGACGTAGGAGATATACCATGAAAAAGAAAAACATTTATTCAATTCGTAAACTAGGTGTAGG
15bchkF	Reverse primer for SpAED precursor, for *in vitro* translation	GATCCTCGAGCATATGGCTGCC

Supplementary Table 2: Strains and plasmids used in this study.

**Plasmid: Name/designation**	**Genotype/Description**	**Source/References**
pWWW412 (vector)	pOS1 vector with an in-built *hprK* promoter	(1)
p*pepV*	pWWW412 carrying *pepV*	Laboratory collection
p*secA*$_{\text{TW-STREP}}$ (pOL3)	pWWW412 carrying *secA*$_{\text{TW-STREP}}$	Laboratory collection
p*secA* (pOL1)	pWWW412 carrying *secA*	Laboratory collection
pOL4	pET15b-*secA-H6*	Laboratory collection
pSY273	pET16b-*H6-pepV*	Laboratory collection
pKOR1	An *E. coli-S. aureus* shuttle vector for allelic replacement	(2)
**Strains: Name/designation**		
Wild type (WT)	*S. aureus* RN4220 *sau1 hsdR*, a derivative of NCTC8325-4	(3, 4)
Δ*spa*Δ*sbi* (WY110)	RN4220 Δ*spa*Δ*sbi*	(5)
Δ*pepV* (SA2101)	RN4220 Δ*pepV*	This study
Δ*spa*Δ*sbi*Δ*pepV* (SA2102)	RN4220 Δ*spa*Δ*sbi*Δ*pepV*	This study
SEJ1	RN4220 Δ*spa*	(6)

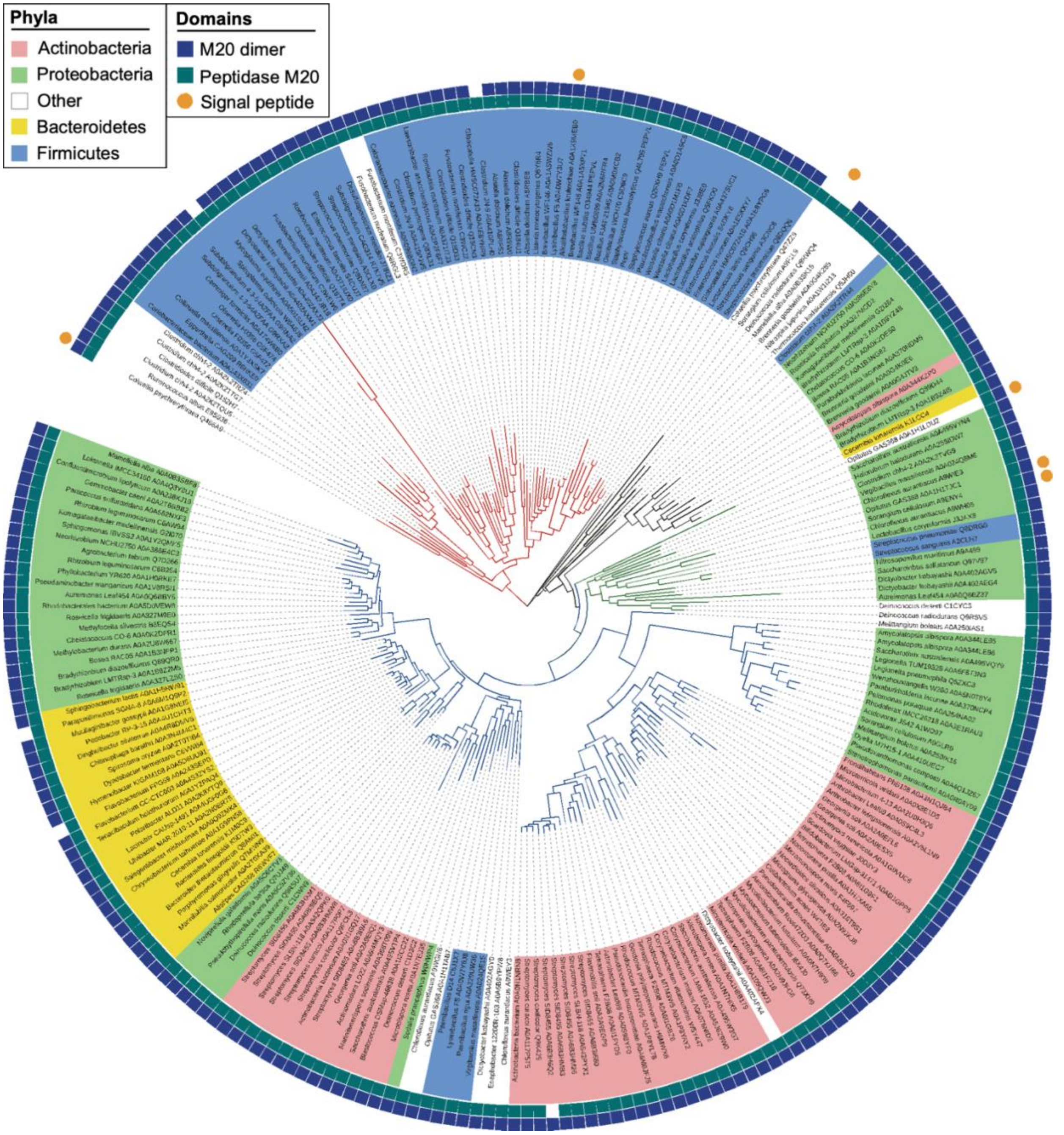

Supplementary Figure 1: A phylogenetic tree of PepV proteases was generated using the SHOOT tool (7) to identify four clades (red, black, green, blue) from the following phyla: Actinobacteria (pink), Proteobacteria (green), Bacteriodetes (yellow), Firmicutes (blue) and others (white). Outer circles indicate the presence of the Pepdiase M20 domain (green squares), dimerization M20 domain (blue squares) and predicted signal sequence (gold circles). Domain annotation was sourced from the InterPro database and visualized using the iToL phylogeny visualization tool (8, 9).

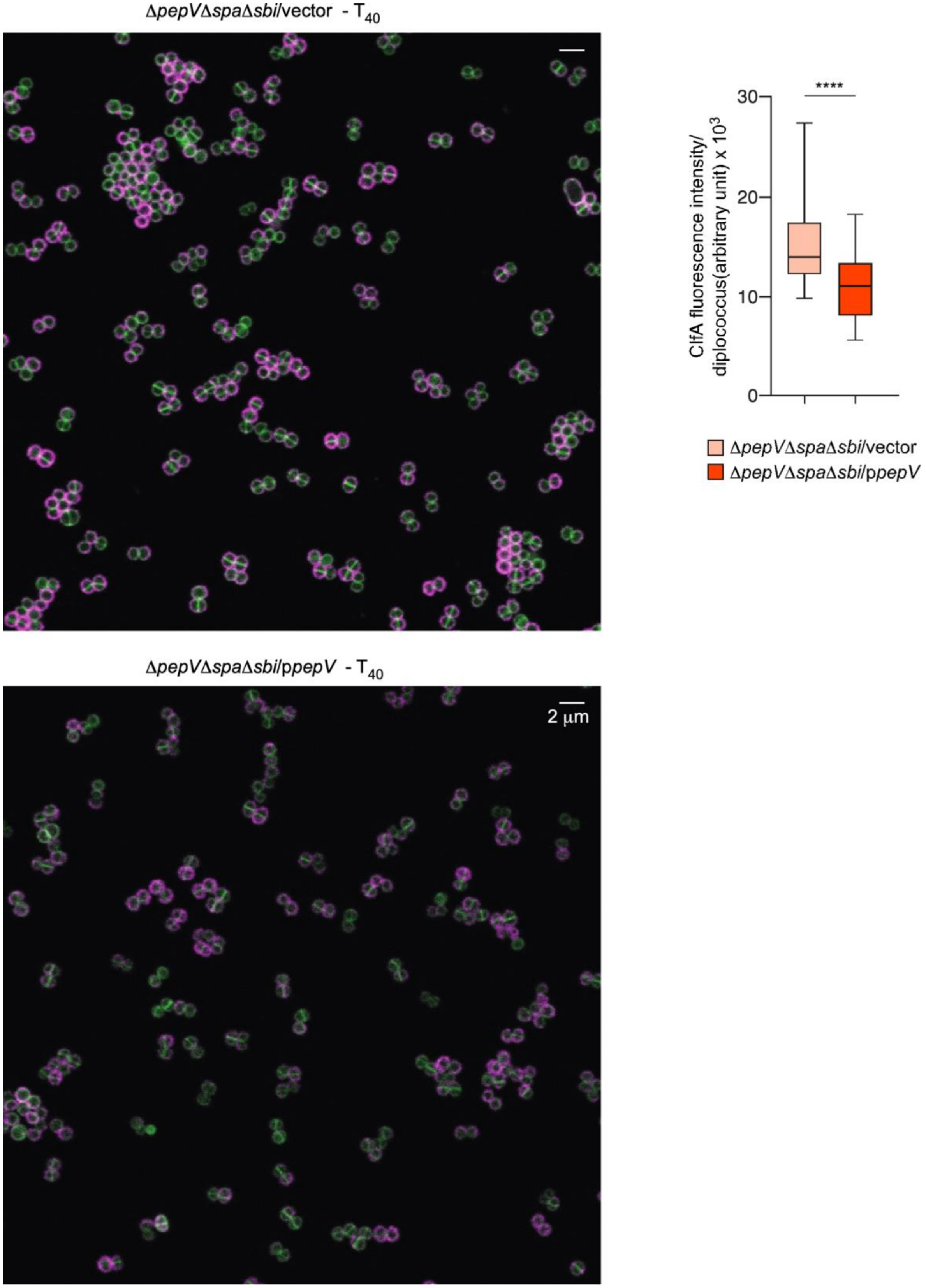
ΔpepVΔspaΔsbi/vector - T40
ΔpepVΔspaΔsbi/ppepV - T40
2 μm
ClfA fluorescence intensity/
diplococcus(arbitrary unit) x 10^3
30
20
10
0
****
ΔpepVΔspaΔsbi/vector
ΔpepVΔspaΔsbi/ppepV

Supplementary Figure 2: Reduced ClfA display in the presence of PepV. Representative confocal micrographs of Δ*pepV* bacteria also lacking the immunoglobulin binders (Δ*spa*Δ*sbi*) to enhance ClfA labeling. Top and bottom micrographs show cells carrying an empty vector or plasmid encoded *pepV* (p*pepV*), respectively Cells underwent trypsin treatment to eliminate surface-exposed proteins followed by 40-minute incubation with trypsin inhibitor (T_{40}) to monitor newly synthesized ClfA. Samples were incubated with BODIPY-FL vancomycin (green) to stain the bacterial cell wall and with ClfA-specific monoclonal followed by Alexa Fluor 594-conjugated secondary antibodies (magenta). Quantification of ClfA immune signals is shown to the right.

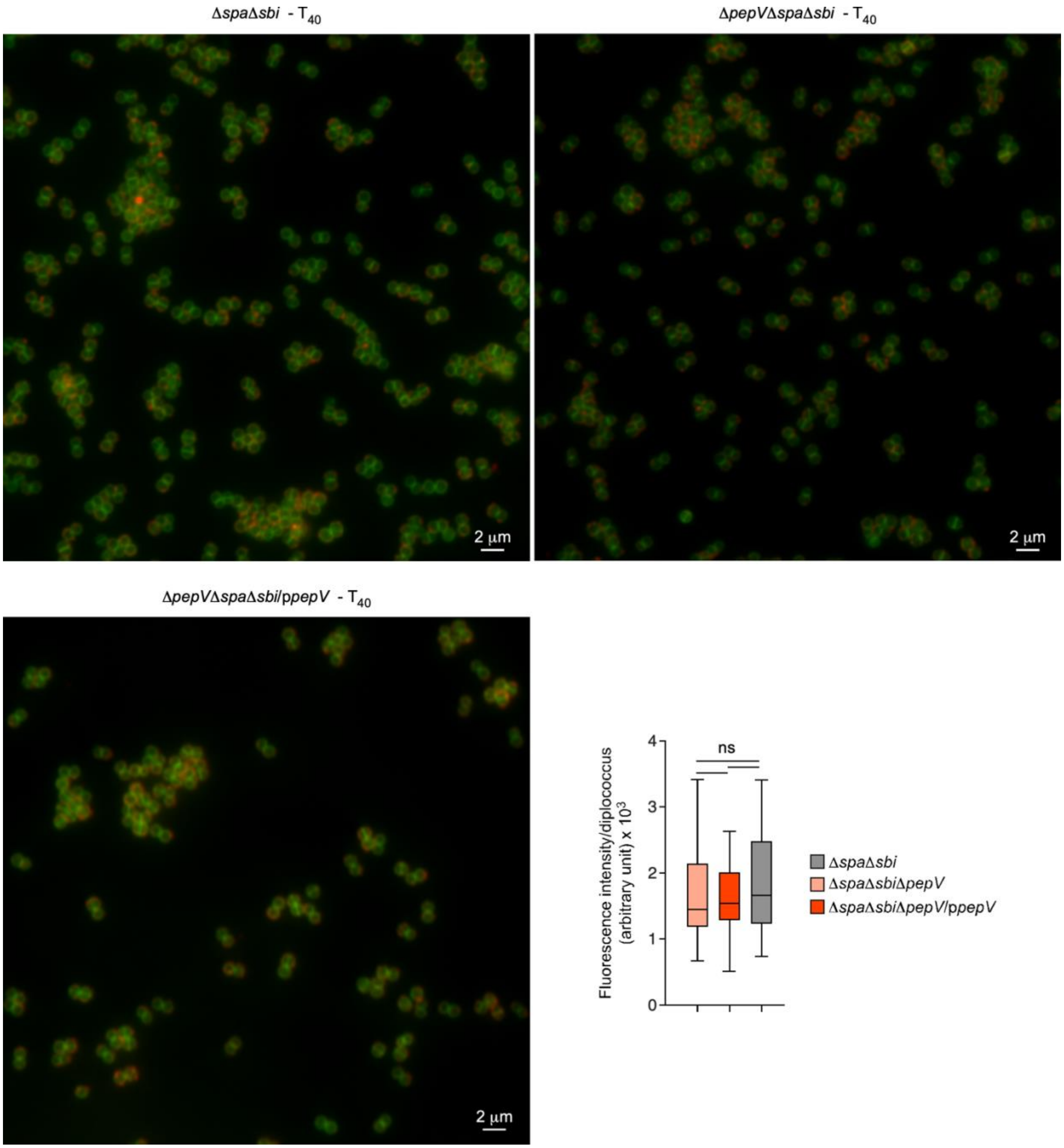

Supplementary Figure 3: PepV does not alter the surface display of SasF, a sortase A anchored protein that lacks a YSIRK motif. Representative confocal micrographs of bacteria lacking the immunoglobulin binders and otherwise wild type (top left; Δ*spa*Δ*sbi*), lacking *pepV* (top right; Δ*pepV* Δ*spa*Δ*sbi*) and a complemented strain (bottom; Δ*pepV* Δ*spa*Δ*sbi*/p*pepV*). Cells underwent trypsin treatment to eliminate surface-exposed proteins followed by 40-minute incubation with trypsin inhibitor (T_{40}). Samples were incubated with BODIPY-FL vancomycin (green) to stain the bacterial cell wall and with

SasF-specific monoclonal followed by Alexa Fluor 594-conjugated secondary antibodies (red). Quantification of SasF immune signals did not reveal any statistical difference between the three strains.

REFERENCES

1. Bubeck Wardenburg J, Williams WA, Missiakas D. 2006. Host defenses against *Staphylococcus aureus* infection require recognition of bacterial lipoproteins. Proc Nat Acad Sci USA 103:13831-13836.

2. Bae T, Schneewind O. 2006. Allelic replacement in Staphylococcus aureus with inducible counter-selection. Plasmid 55:58-63.

3. Nair D, Memmi G, Hernandez D, Bard J, Beaume M, Gill S, Francois P, Cheung AL. 2011. Whole-genome sequencing of *Staphylococcus aureus* strain RN4220, a key laboratory strain used in virulence research, identifies mutations that affect not only virulence factors but also the fitness of the strain. J Bacteriol 193:2332-5.

4. Kreiswirth BN, Lofdahl S, Betley MJ, O'Reilly M, Schlievert PM, Bergdoll MS, Novick RP. 1983. The toxic shock syndrome exotoxin structural gene is not detectably transmitted by a prophage. Nature 305:709-12.

5. Yu W, Missiakas D, Schneewind O. 2018. Septal secretion of protein A in Staphylococcus aureus requires SecA and lipoteichoic acid synthesis. Elife 7.

6. Grundling A, Schneewind O. 2007. Synthesis of glycerol phosphate lipoteichoic acid in Staphylococcus aureus. Proc Natl Acad Sci U S A 104:8478-83.

7. Emms DM, Kelly S. 2022. SHOOT: phylogenetic gene search and ortholog inference. Genome Biology 23:85.

8. Paysan-Lafosse T, Blum M, Chuguransky S, Grego T, Pinto BL, Salazar Gustavo A, Bileschi Maxwell L, Bork P, Bridge A, Colwell L, Gough J, Haft Daniel H, Letunić I, Marchler-Bauer A, Mi H, Natale Darren A, Orengo Christine A, Pandurangan Arun P, Rivoire C, Sigrist CJA, Sillitoe I, Thanki N, Thomas PD, Tosatto SCE, Wu Cathy H, Bateman A. 2022. InterPro in 2022. Nucleic Acids Research 51:D418-D427.

9. Letunic I, Bork P. 2021. Interactive Tree Of Life (iTOL) v5: an online tool for phylogenetic tree display and annotation. Nucleic Acids Res 49:W293-W296.
